# Hematospermia as a manifestation of severe hypertension in a young man

**DOI:** 10.1002/ccr3.849

**Published:** 2017-03-02

**Authors:** Maria Amasanti, Cleo Huang, Ben Lovell

**Affiliations:** ^1^ Whittington HospitalMagdala AvenueLondonN195NFUK

**Keywords:** Diagnosis, hematospermia, hypertension, urological disease

## Abstract

Whilst hematospermia may indicate urological disease, it is a rare manifestation of severe systemic hypertension. We describe the case of a young man with hematospermia associated with a blood pressure of 228/135. Blood pressure treatment led to resolution of his hematospermia. Physicians must be aware of this potential association.

## Introduction

Hematospermia may occur as a presenting symptom to primary care doctors, emergency doctors, and urologists. It is an alarming symptom for patients, yet may be under‐reported due to patient embarrassment. In young patients, it is almost never a sign of urological malignancy, and usually points toward a self‐limiting disease, such as prostatitis, urethritis, or epididymo‐orchitis 1.

A review of guidelines in the medical literature reveals a focus on urological investigations, such as prostate imaging and biopsy 2, but rarely recommends measurement of the systemic blood pressure, despite severe uncontrolled hypertension being known to be an etiological factor in hematospermia 3. This simple, noninvasive test may negate the need for more invasive investigations and identify an otherwise silent cardiovascular pathology that may require urgent treatment.

## Case Report

A 28‐year‐old Caucasian man living in London presented at a walk‐in GP clinic reporting four episodes of hematospermia over 10 days. In the first three episodes, the patient reported the semen being brown in color. On the fourth episode, there were distinct streaks of red blood within the semen.

The man was heterosexual, and this was his first and only sexual partner. His partner had no history of sexually transmitted infections (STI). The couple was sexually active and using barrier method contraception for family planning reasons.

The patient was otherwise fit and well with no past medical history apart from mild childhood asthma. He had no headaches or changes in vision. He had no cardiac or respiratory symptoms. There was no history of recent trauma. There were no changes in urinary or bowel habits. He had no pain, fever, sweats, weight loss, or fatigue. He had not travelled abroad recently. The patient had never smoked or taken illicit substances. His diet was reasonably healthy, and he walked approximately 5 km a day. He consumed approximately 32 units of alcohol per week and recognized that this as in excess of medical guidelines. There was no family history of inheritable diseases.

On examination, he was comfortable at rest and clinically euvolemic. His abdomen was soft and nontender. His kidneys were not palpable. There were no renal bruits. There was no enlargement or masses in the scrotum. A digital rectal examination was normal with no enlargement or nodularity of the prostate. Respiratory examination revealed normal breath sounds. Examination of the central and peripheral nervous system was entirely normal.

On cardiovascular examination, heart sounds were normal. There was no peripheral edema, and the jugular venous pressure was not raised. There was no radio‐femoral delay, and fundoscopy was normal. His blood pressure was note to be 228/135 mmHg, and his heart rate was 90 beats per minute and regular. Pulse volume and character were normal. His oxygen saturations were 98% on room air. There were no signs of acromegaly or Cushing's syndrome.

Urinalysis was completely negative. His electrocardiogram (ECG) showed normal sinus rhythm. There was no left ventricular hypertrophy, axis deviation, or strain pattern.

His full blood count was as follows: hemoglobin 174 g/L, platelets 234 × 10^9^/L, white blood count 5.9 × 10^9^/L, and mean corpuscular volume was 85 fl. His CRP was <1 mg/L. His urea and electrolytes were sodium 142 mmol/L, potassium 4.3 mmol/L, urea 4.7 mmol/L, and creatinine 96 mmol/L. His estimated glomerular filtration rate was slightly reduced at 84 mL/min. His chest radiograph was normal. Ultrasound and computerized tomography of the urinary tract were normal.

Twenty four‐hour urinary metanephrines and a serum renin–aldosterone level were with normal range, ruling out pheochromocytoma and hyperaldosteronism as causes of hypertension, respectively. An echocardiogram was entirely normal. More specifically, there was no left ventricular hypertension nor diastolic dysfunction, which would be the expected findings in a case of chronic hypertensive heart disease.

A diagnosis of hematospermia secondary to severe essential hypertension was made. There were no clinical signs of malignant hypertension. The absence of other urological symptoms made inflammation or infection of the urinary tract unlikely, and the lack of proteinuria or left ventricular hypertrophy on ECG implied that the hypertension was of recent rather than chronic onset.

The patient was treated with an angiotensin‐converting enzyme inhibitor (ACE‐i), per National Institute for Health and Care Excellence guidelines. A dosage of 2.5 mg of ramipril daily was sufficient to control his blood pressure.

The patient purchased a home blood pressure machine and monitors his blood pressure daily. His systolic pressure lowered steadily to an average of 165 mmHg. Normotension led to complete resolution of the hematospermia.

## Discussion

Hematospermia is almost always a benign and transient phenomenon 4, 5, 6. It is most commonly a sequelae of infection, such as prostatitis, urethritis, epididymo‐orchitis; medical intervention, such as a prostate biopsy; or trauma, such as a perineal injury. Infective causes may be due to sexually transmitted diseases, or urinary tract infections, and respond readily to a short course of oral antibiotics. Hematospermia associated with infection of the lower urinary tract is usually associated with painful ejaculation.

Hematospermia is only rarely associated with prostatic malignancy. Cohort studies have described an incidence of 0.5% in cases of prostate cancer 7. Reg flags for potentially sinister causes of hematospermia are patient's age over 40, recurrent or persistent hematospermia, risk factors for prostate cancer, such as family history or Afro‐Caribbean ethnicity, and constitutional symptoms, such as weight loss, anorexia, or bone pain 1. In the absence of such red flags, and if no causative factor is identified, most authorities recommend a “watch and wait” policy, and reassure the patient that the symptoms will vanish within days or weeks 2, 6, 7. Rarer causes of hematospermia include coagulopathy, schistosomiasis, prolonged sexual intercourse, and malignancies of the testis, urethra, and bladder 1.

Severe hypertension is a recognized but rare cause of hematospermia 5.We have identified only three case reports in the existing literature describing this correlation 8, 9, 10. This may be due to the rarity of such a phenomenon, under‐recognition of hypertension in patients with hematospermia, or linked to a general under‐reporting of transient hematospermia by patients 11.

Severe essential hypertension in young patients is uncommon. It is crucial to diagnose it early, as prolonged hypertension places an otherwise healthy patient at risk of end‐organ damage. Hypertensive urgency – or malignant hypertension – is a dangerous condition that may lead to irreversible damage to the central nervous system, retinas, or the kidney, but many cases are asymptomatic. It is therefore feasible that a young man could present to a physician with hematospermia and no other signs or symptoms of disease, and not have his BP measured as part of the assessment. This case is therefore an important lesson in the initial clinical workup of this symptom; the hematospermia is a benign and temporary occurrence, but the underlying blood pressure abnormality is potentially serious and requires urgent management.

## Conclusion

Hematospermia in young males without other red flag symptoms is almost exclusive benign and self‐limiting. Whilst uncontrolled hypertension in this cohort is uncommon, consideration of severe hypertension as a cause of hematospermia must be considered by the treating physician, as correction of the abnormal blood pressure will be the priority consideration.

## Conflict of Interest

None declared.

## Authorship

MA: wrote the main body of the case report. CH: reviewed the manuscript and made material changes and revisions. BL: critically appraised the manuscript content and made minor revisions.
